# Distinct Pattern of NPY in Gastro–Entero–Pancreatic System of Goat Kids Fed with a New Standardized Red Orange and Lemon Extract (RLE)

**DOI:** 10.3390/ani11020449

**Published:** 2021-02-09

**Authors:** Elena De Felice, Daniela Giaquinto, Sara Damiano, Angela Salzano, Simona Fabroni, Roberto Ciarcia, Paola Scocco, Paolo de Girolamo, Livia D’Angelo

**Affiliations:** 1 School of Biosciences and Veterinary Medicine, University of Camerino, Via Pontoni 5, 62032 Camerino, Italy; elena.defelice@unicam.it (E.D.F.); daniela.giaquinto@unicam.it (D.G.); paola.scocco@unicam.it (P.S.); 2 Department of Veterinary Medicine and Animal Productions, University of Naples Federico II, 80137 Naples, Italy; sara.damiano@unina.it (S.D.); angela.salzano@unina.it (A.S.); roberto.ciarcia@unina.it (R.C.); livia.dangelo@unina.it (L.D.); 3 Research Centre for Olive, Fruit and Citrus Crops, Council for Agricultural Research and Economics (CREA), 95024 Acireale, Italy; simona.fabroni@crea.gov.it

**Keywords:** gastro-intestinal apparatus, feed additive, small ruminants, goats, neuropeptides, natural compound, polyphenols

## Abstract

**Simple Summary:**

In the last decades the European ban towards antibiotics resulted in an increase of the number of studies on the effects of natural feed additives, that can enhance the health of farm animals intended for human consumption. Polyphenols such as flavanones and anthocyanins (responsible of the red, purple or blue colors) are bioactive compounds found in fruits and vegetables. Polyphenols possess multiple pharmacological characteristics, like antioxidant, anti-inflammatory and immunostimulant properties. Although many of the biological effects of polyphenols are known, only a limited number of studies has been focused on the effects of their supplementation in ruminant diet. Therefore, we evaluated the effect of a diet supplemented with a standardized powder extract, red (blood) orange and lemon extract (RLE), rich in flavanones, anthocyanins and other polyphenols on the neuropeptide Y (NPY) distribution in the gastro–entero–pancreatic system of goat kids. In mammals, NPY occurs in both the central and peripheral nervous systems and it is involved in the control of different physiological processes, including food intake regulation. For the first time, we document that NPY is widely distributed in the abomasum, duodenum and pancreas of goat kids and that significantly increases in the abomasum and pancreas of RLE supplemented feed animals.

**Abstract:**

The use of natural compounds as feed additive is also increasing in farm animals, thanks to the beneficial effect on both animals and consumers health. Here, we questioned whether natural extracts, such as red orange and lemon extract (RLE) rich in flavanones, anthocyanins, and other polyphenols, used as feed additives could display an effect on the neuropeptide Y (NPY) in the gastro–entero–pancreatic tract of goat kids. NPY is one of the most abundant neuropeptides in mammals, known for its orexigenic role although it is involved in many central and peripheral functions. We carried out immunohistochemical analyses on samples of abomasum, duodenum and pancreas collected from two experimental groups: one fed with standard diet and one with standard diet + RLE. For the first time we document NPY distribution in the abomasum, duodenum and pancreas of goats and observe the highest number of NPY positive cells in neuroendocrine cells of duodenum. Remarkably, upon RLE feed supplementation, NPY immunoreactive cells increased significantly in abomasal epithelium and pancreatic islets but not in duodenum, likely due to pH variation of abomasum and duodenum. Our observations represent a baseline for future studies on the interaction between neuropeptides and polyphenols, used as feed additive.

## 1. Introduction

The use of natural compounds as feed supplementation has been facing a new era, especially since the European ban on ionophore antibiotics (European Commission, Directive 1831/2003/CEE, 2003). For many years antibiotics’ employ has been a usual praxis in livestock farming, to augment growth, enhance feed conversion efficiency and prevent and treat diseases [1]. However, the utilization of antibiotics has amplified the extent of antimicrobial resistance, posing severe warnings to both animal and human health and food security [2].

Recently, among natural compounds there has been a growing interest in the consumption of anthocyanin-rich food. Anthocyanins (ANTs), which belong to the flavonoid family, are water soluble polyphenolic pigments widespread in the plant kingdom, responsible of the blue, purple, red and orange pigmentation of various vegetables and fruits [3]. ANTs have been demonstrated to have numerous benefits, such as anti-inflammatory, antioxidant, anti-obesity, anti-angiogenesis, anti-cancer, anti-diabetes, anti-microbial, anti-proliferative, neuroprotection and immunomodulation properties [4]. The anti-inflammatory and antioxidant properties have been demonstrated by inducing the downregulation of cyclooxygenases-2 and the inhibition of prostaglandin E2 production [5] and by decreasing the activation of NF-kB transcription factor [6]. ANTs have been shown to contrast oxidative stress directly, donating or transferring electrons from hydrogen atoms [7] and indirectly through the activation of specific detoxification enzymes [8]. Recent studies documented a beneficial effect of ANTs also on gut microbiota populations, acting as prebiotics. In particular the consumption of food rich in ANTs is associated with the increase in the large intestine of *Bifidobacterium* spp. and *Lactobacillus* spp., two Gram-positive species beneficial in the treatment of diarrhoea and other intestinal diseases [4,9,10]. Very recently, the value of ANTs and proanthocyanidin as feed supplementation has been investigated and proposed as ruminant feedstuffs because of the positive effects on ruminal degradation, as well as antioxidant contents and activities [11,12]. Anthocyanin-rich purple corn (*Zea mays* L.) showed a lowering effect on aminotransferase activity with concomitant enhancement of superoxide dismutase (SOD) activity in the plasma of lactating dairy cows [11], while in dairy goats it improved SOD activity in plasma and up-regulated nuclear factor (erythroid-derive 2)-like 2 gene expression in mammary gland [13]. However, ANTs were found to have poor palatability owing to their bitter taste [14], with consequences on food consumption and food intake behaviour.

Despite the great number of papers devoted to characterize the disparate biological effects of ANTs and flavanones (FLAVs), only a limited number of studies have so far focused on the potential effect of natural compound rich in ANTs and FLAVs for the diet supplementation in farm animals. FLAVs and hydroxycinnamic acids are currently used for the treatment of capillary fragility and for other diseases whose aetiology is linked to the disruptive action of free radicals [15,16,17,18,19]. Recently, it has been demonstrated that a lemon fruit extract rich in eriocitrin and other FLAVs can positively act in the regulation of adipocyte differentiation and lipid accumulation [20]. 

Neuropeptide Y (NPY) is a 36-amino acid peptide widely distributed in both the central and peripheral nervous systems and it has been functionally related to regulation of feeding behaviour and gastrointestinal tract motility [21]. It belongs to the neuroendocrine peptide NPY family, which also includes peptide YY (PYY) and pancreatic polypeptide, mainly occurring in the gastro-enteric tract of all mammals. In goats, NPY is localized at 75, 95 and 113 days of gestation in the lamina propria-submucosa, tunica muscularis and myenteric of the three prestomachs [22]. In the abomasum, NPY was detected at 75 days of gestation [23]. Remarkably, compared to sheep and cow, NPY is detected earlier in goats [22,23]. However, there are no more data on the occurrence and regulation of NPY in the postnatal glandular stomach of goats neither in the duodenum nor in the pancreas.

Recently, it has been demonstrated that ANTs have the capability to reduce body weight and food intake through their modulatory effect on NPY and GABAB_1_R in rat hypothalamus [24]. In the rat hypothalamus, the consumption of fruits containing ANTs induced an increase of NPY mRNA expression in non-obese animals [25]. Knowledge on the peripheral regulation of NPY upon ANTs and FLAVs supplemented feeding is still lacking, although it has well established that ANTs are bioavailable and metabolized at gastro-intestinal level, as demonstrated in weanling pigs [26]. Therefore, we propose in this study to investigate whether RLE may regulate the occurrence of NPY protein in the abomasum, duodenum and pancreas of goat kids.

We therefore undertook this study to investigate the effect of a red orange and lemon extract (RLE) rich in FLAVs, ANTs and other polyphenols, on the gastro-enteric regulation of NPY in goat kids from the birth until weaning. 

## 2. Materials and Methods 

### 2.1. Experimental Design and Animals 

The research was approved with Protocol PG/2019/0028161 of 19 March 2019 by the Animal Welfare Body of the University of Naples Federico II. The experimental procedures were carried out in the experimental farm of the Council for Agricultural Research and Economics, Research Center for Animal Production and Aquaculture (CREA-ZA, Potenza, Italy). 

The standardized powder phytoextract rich in flavanones, anthocyanins, and other polyphenols was obtained by a patented extraction process (Italian Patent No. 102017000057761) from blood orange and lemon processing wastes (red orange and lemon extract, here named RLE). The standardized extract was realized in the laboratories of CREA-Research Centre for Olive, Fruit and Citrus Crops (CREA-OFA, Acireale, Italy) for research purposes only. Information concerning RLE chemical composition are shown in Table 1. Identification and relative concentrations of individual flavanones and anthocyanins are reported in previous works [19,20,21,22,23,24,25,26,27]. 

Sixty kids of Saanen bred, males and females, after colostrum administration, were randomly divided into two homogenous (homogeneous for body weight, sex, age and physiological condition) groups (named Control and Treated respectively) of 30 kids each and housed in single boxes. The numerousness of animal for each group was calculated and considered optimal for a significance level of 0.05, a test power of 0.9 and an effect size of 0.85.

The two experimental groups were fed for the whole experimental period (40 days) with: (1) standard diet made of hay (100 g) and kids starter (150 g) (CTRL group); (2) standard diet supplemented with RLE (90 mg/kg) (TRT group). The dose of RLE extract (90 mg/kg of live weight) was defined according to a previous work [27]. Kids were weighed daily in order to record the average weight gain and RLE extract was mixed with water to obtain a cream [28], which was then administered through a syringe directly in the mouth.

All kids underwent natural suckling throughout the experimental period. Water was provided *ad libitum* and the diets were wet (water to feed ratio 3:1). Data about daily average weight gain were collected. At the end of the experimental period animals were slaughtered at a public abattoir in accordance with the Art. 29 of the Council Regulation (EC) No. 1099/2009 on the protection of animals at the time of killing.

### 2.2. Tissue Sampling

Six samples of abomasum, duodenum (removed 4 cm from the pyloric sphincter) and pancreas were collected from subjects of each group. The samples were promptly immersed in 10% neutral-buffered formalin solution for 24 h and so carefully fixed [29]. All specimens were then dehydrated with a graded ethanol series, cleared in xylene, embedded in paraffin wax and cut into 7-μm-thick serial sections. The sections were stained with hematoxylin and eosin for the morphological analysis. 

### 2.3. Single Immunostaining

After deparaffinization, the sections were incubated with 3% hydrogen peroxide for 30 min at room temperature (RT) to block endogenous peroxidase activity, and then were rinsed in 0.1 M phosphate-buffered saline (PBS), pH 7.4 for 15 min, subsequently pre-incubated for 1 h at RT with the blocking solution (cat# n191356, MP biomedical LLC, Solon, OH, USA) (1:5 in 0.01 M PBS). The sections were then incubated with polyclonal antibody raised in rabbit against NPY (1:500, cat# ab30914, Abcam, Cambridge, UK) and polyclonal antibody raised in rabbit against 5-HT (1:5000, cat# 20080, Immunostar, Hudson, Wisconsin, WI, USA), overnight at 4 °C in humid chamber. The day after, the sections were rinsed in PBS for 15 min and incubated for 30 min at RT with ultrapolymer cocktail (cat# UNIHRP-015, ImmunoReagents, Inc., Raleigh, NC, USA). Immunoreactive sites were visualized using a fresh solution of 10 mg of 3–3′ diaminobenzidine tetrahydrocloride (DAB, cat# D5905, Sigma-Aldrich, Darmstadt, Germany) in 15 mL of 0.5 M Tris buffer, pH 7.6, containing 0.03% hydrogen peroxide.

#### Double Immunostaining

Double immunostaining of NPY and 5-HT was performed according to Wessel & McClay [30]. After dewaxing, the sections were rinsed in 0.1 M PBS for 10 min and pre-incubated for 1 h at RT with the blocking solution, and then incubated with the first primary antiserum 5-HT (1:500) for 36 h at 4 °C in humid chamber. Then the sections were washed in PBS and incubated with goat anti-rabbit Fab fragment conjugated to tetramethylrhodamine-5-(and 6) isothiocyanate fluorochrome (1:30, cat# 111-297-003, Jackson ImmunoResearch, Cambridge, UK) for 2 h at 37 °C. Thereafter, the sections were rinsed in PBS and incubated with NPY (1:50) over night at 4 °C in humid chamber. After rinsing in PBS, the sections were treated with affinity-pure goat anti-rabbit IgG conjugated to fluoroscein isothiocyanate fluorochrome (1:50, cat# 111-095-006, Jackson ImmunoResearch, Cambridge, UK) for 2 h at 37 °C. Finally, the sections were washed with PBS and mounted.

### 2.4. Controls of Specificity

Positive controls were made by sections of mouse duodenum (Supplementary Figure S1). Internal reaction controls were carried out by substituting primary antisera or secondary antisera with PBS or normal serum in the specific step.

### 2.5. Image Acquisition

Fluorescent and light images were observed and analyzed by Nikon Eclipse 90i. The digital raw images were optimized for image resolution, contrast, evenness of illumination, and background by using Adobe Photoshop CS5 (Adobe Systems, San Jose, CA, USA). For cell counting, micrographs were saved in TIFF format and adjusted for light and contrast.

### 2.6. Cell Counting

In order to evaluate the number of NPY containing cells, six random sections of abomasum, intestine and pancreas for each group, composed of an equal number of males and females, were selected. Positive cells were counted in ten randomly selected observation fields per section (observation field was equal to 26.67 × 20.00 inches; 1920 × 1440 pixels; RGB 11 MB; microscopy magnification 20×. Therefore, 60 observation fields were evaluated for each sample from each animal. The obtained data were pooled and analysed as comparison of mean value [31]. Statistical analysis was performed using Student’s t-test (GraphPad Prism v. 3.0, GraphPad Software Inc., San Diego, CA, USA). The differences were considered statistically significant at *p* ˂ 0.05.

## 3. Results

Any difference was recorded in terms of daily average weight gain that was 128 ± 0.01 vs. 113 ± 0.01 g, respectively for group TRT and CTRL.

### 3.1. Distribution of NPY and 5-HT in the Abomasum, Duodenum and Pancreas of Control and Treated Animals

**Figure 1:**
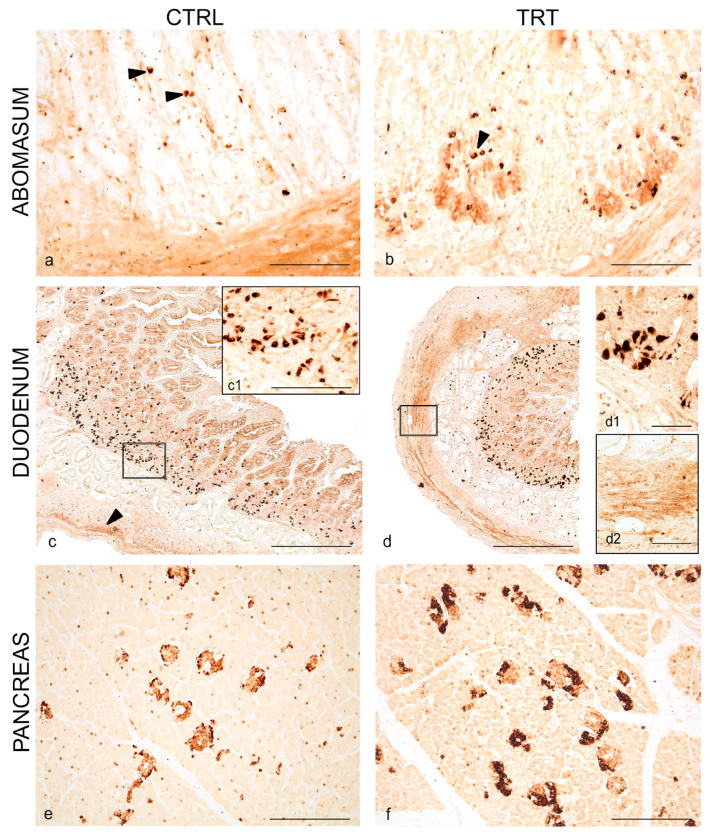
Neuropeptide Y (NPY) immunoreactivity in the abomasum, duodenum and pancreas of control and treated goat kids. Scattered positive cells (arrowheads) in the epithelium of gastric mucosa of control (**a**) and anthocyanins (ANTs) fed animals (**b**). Overview of NPY distribution in neuroendocrine cells (**c**) in the crypt of Lieberkühn and varicose positive fibers (arrowhead) in the muscular layer of duodenum of control (**a**) and ANTs fed animals (**d**). Higher magnification of neuroendocrine cells in the crypt of Lieberkühn (**c1**,**d1**) and fibers in the muscular layer (**d2**). Overview of NPY distribution in the pancreatic islets of control (**e**) and ANTs fed animals (**f**). Scale bar: (a,b,e,f) = 50 µm; (c,d) = 100 µm; (c1,d1,d2) = 25 µm.

Immunoreactivity to NPY was observed in all analyzed tracts of the digestive apparatus of kids, in both experimental groups. In the abomasum, NPY was seen in scattered cells of the epithelium (Figure 1a), which appeared more abundantly distributed, also at the basis of epithelium, in treated animals (Figure 1b). Immunoreactivity to NPY was also seen in varicose fibers in the muscular layer of the abomasum in both groups (Figure 1a,b).

In the duodenum, NPY immunostaining was visualized in numerous cells in the crypt of Lieberkhϋn in animals of both groups (Figure 1c,c1,d,d1). NPY immunoreactivity was seen in varicose fibers of muscular layer of the duodenum and appeared weaker in control animals (Figure 1c) compared to treated animals (Figure 1d,d2). 

In the pancreas, numerous NPY immunoreactive cells were seen in the pancreatic islets of control and treated animals (Figure 1e,f), displaced at the margin of the islet. Some scattered positive NPY cells were also seen over the pancreatic parenchyma. 

**Figure 2:**
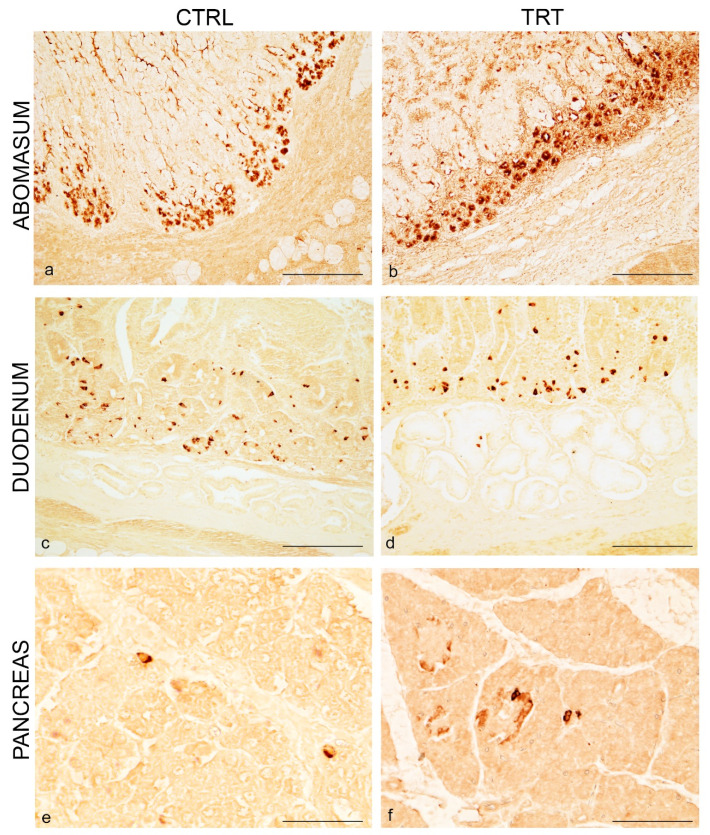
5-HT immunoreactivity in the abomasum, duodenum and pancreas of control and treated goat kids. Numerous positive cells in the glands at the basis of gastric epithelium of control (**a**) and ANTs fed animals (**b**). Overview of 5-HT distribution in neuroendocrine cells (**c**) in the crypt of Lieberkühn of duodenum of control (**c**) and ANTs fed animals (**d**). Overview of 5-HT distribution in scattered cells in the pancreatic parenchyma of control (**e**) and ANTs fed animals (**f**). Scale bar: (a,b,f) = 50 µm; (c,d) = 100 µm; (e) = 25 µm.

To better characterize NPY immunoreactive cells we conducted immunohistochemical experiments by using 5-HT as classical neuroendocrine marker. In single staining, immunoreactivity to 5-HT was clearly seen in all tracts, although to a less extent in the pancreas. In the abomasum immunoreactivity to 5-HT was seen in cells of the abomasal glands displaced at the basis of the epithelium of control and treated animals (Figure 2a,b). 

In the duodenum, 5-HT immunostaining was observed in cells in the crypt of Lieberkhϋn in animals of both groups (Figure 2c,d).

In the pancreas, immunoreactivity to 5-HT was seen in scattered and isolated cells over the pancreatic parenchyma in the two experimental groups (Figure 2e,f).

### 3.2. Co-Localization of NPY and 5-HT in the Abomasum, Duodenum and Pancreas 

We then performed experiment of immunofluorescence on sections of treated animals to evaluate whether NPY was co-localized with 5-HT. 

**Figure 3:**
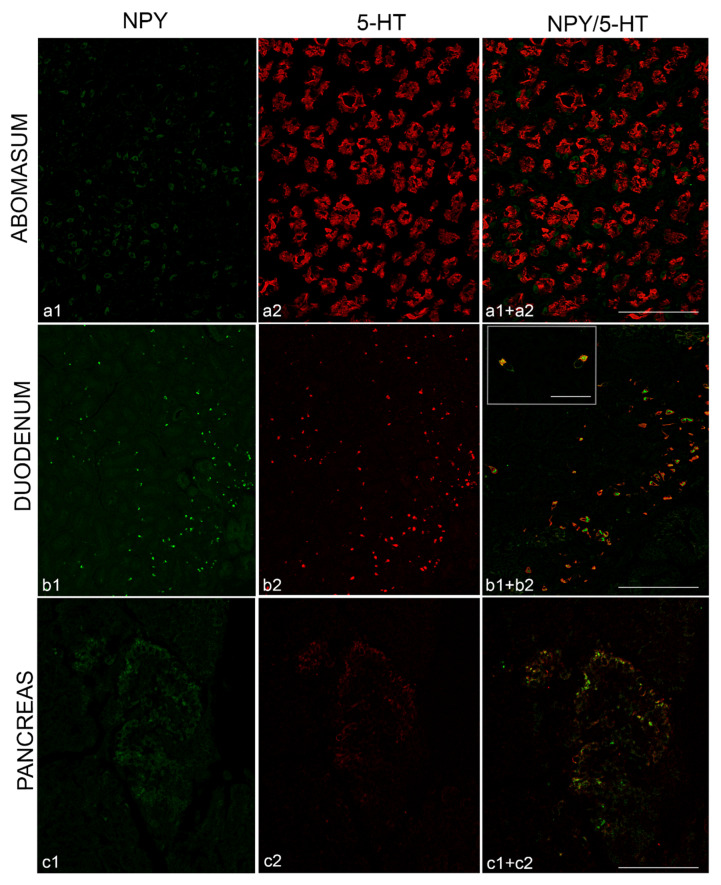
Immunofluorescence of NPY (green) and 5-HT (red) in the abomasum, duodenum and pancreas of goat kids fed with red orange and lemon extract (RLE) supplementation. NPY distributed in sparse cells of abomasal epithelium (**a1**) and 5-HT in glands at the basis of gastric epithelium (**a2**). Absence of NPY/5-HT co-localization (**a1+a2**). NPY (**b1**) and 5-HT (**b2**) co-distributed in the neuroendocrine cells of duodenum, with NPY mainly localized in the cytoplasm and 5-HT on the cellular membrane (**b1+b2**). NPY (**c1**) and 5-HT (**c2**) in the pancreatic islet, localized in different cells (**c1+c2**). Scale bar: (a) = 25 µm; (b,c) = 50 µm.

In the abomasum, NPY and 5-HT displayed a clear different distribution, with NPY distributed in scattered cells of the epithelium and at the basis of epithelium while 5-HT abundantly distributed in cells of glands (Figure 3a). In the duodenum all cells immunopositive to NPY were co-localized with 5-HT (Figure 3b). Remarkably, NPY and 5-HT in the same cells displayed a different localization: the former in the cytoplasm and the latter on the cytoplasmic membrane (Figure 3b). In the pancreas, any co-distribution was seen between NPY and 5-HT, because NPY was distributed in the pancreatic islets while 5-HT was scattered along the pancreatic parenchyma (Figure 3c). 

### 3.3. NPY Cell Distribution

**Figure 4:**
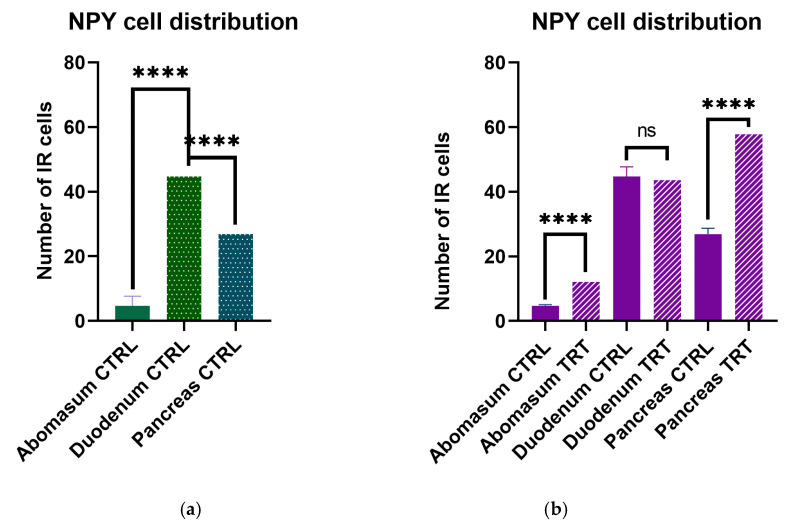
Number of immunoreactive cells in the gastro–entero–pancreatic system of goat kids. (**a**) Higher distribution in the duodenum and pancreas in control animals. (**b**) Statistically increased number of NPY immunoreactive cells in the abomasum and pancreas but not in the duodenum following RLE feed supplementation. **** *p* > 0.0001; ns non-significant.

We counted the number of immunoreactive cells to NPY in abomasum, duodenum and pancreas to quantify the relative distribution in each tract. Statistically significant number of NPY positive cells was counted in the duodenum (*p* > 0.0001) compared to abomasum and pancreas (*p* < 0.05) (Figure 4a). 

To assess if ANTs feed supplementation could have an effect of the regulation of NPY protein, we quantified the number of immunoreactive cells in the three different selected segments. Notably, we observed a strong significantly increase of NPY immunolabeled cells in the abomasum and pancreas (*p* > 0.0001) of ANTs supplemented feed animals while a non-significant increase was observed in the duodenum (*p* < 0.05) of the same animals (Figure 4).

## 4. Discussion

Polyphenols and ANTs have aroused considerable interest because of their high antioxidant, antimicrobial, anti-inflammatory activities in human and animal health [27,32,33]. RLE extracted from citrus and lemon fruits are a rich source of health-promoting compounds. Indeed, pigmented oranges (*Citrus sinensis* L. Osbeck) are particularly rich in anthocyanins (95% of these being represented by cyanidin-3-glucoside and cyanidin-3-6′′-malonyl-glucoside), flavanones (hesperidin, narirutin and didymin) and hydroxycinnamic acids (caffeic, coumaric, sinapic and ferulic acids). Lemon fruit (*Citrus limon* L. Burm) are also rich in flavanones (eriocitrin, hesperidin and diosmin) and other polyphenols. All these compounds exert their biological activity due to their capacity to scavenge free radicals. Increasing demand of food supplements containing relevant amounts of these compounds have been recently recorded for both human and animal healthy diets [34,35]. Dietary consumption of ANTs has revealed benefits in animal performance [36]. The preventive potential of ANTs depends on their absorption and metabolism. Several studies conducted on animal models indicated that the absorption likely starts in the stomach [37] and in the small intestine, mainly in the duodenum and jejunum [38]. Here we investigated whether feeding supplementation of RLE rich in FLAVs, ANTs and other polyphenols had local effect on gastro–entero–pancreatic cells containing NPY. 

For the first time, we document that NPY is widely distributed in the gastro–entero–pancreatic tract of goat kids and its occurrence is regulated by RLE supplemented feed, both in male and female subjects. NPY was observed in scattered cells of the epithelium and in typical varicosity in the muscular layer of the abomasum; in neuroendocrine cells and varicose fibers of the muscular layer in the duodenum and in pancreatic islet cells. The wider distribution was seen in the duodenum, and to a less extent in the pancreas and abomasum. NPY in the abomasum appears already during prenatal development [23] and it seems to contribute to the earlier secretory capacity development compared to sheep and cattle [22]. NPY is known to regulate gastric acid secretion [39] and inhibit water and electrolyte secretion, in particular Cl^−^ secretion, from the small intestine [40]. In addition, NPY serves many other functions in the gastrointestinal system, such as inhibition of motility [41], mediating action between the nervous and immune system [42], regulation of inflammation by recruiting immature dendritic cells and promoting helper T-cell polarization [43]. As expected, we observed NPY in neuroendocrine cells of duodenum epithelium and in the varicose fibers in the muscle layer, accordingly to the vast literature available in mammals [44]. To better characterize NPY containing cells, we co-stained with 5-HT, which is a multifunctional bioamine, derived from the amino acid tryptophan, playing a role in several complex biological functions, such as suppression of appetite; influence of learning, memory and happiness; regulation of sleep and behaviour. Although serotonin has long been recognized for its critical functions in central nervous system development and function, the majority of the body’s serotonin, however, is synthesized in specialized enteroendocrine cells within the gastrointestinal mucosa called enterochromaffin cells, where it plays key roles in enteric nervous system development and function [45]. The main function of 5-HT in the gastrointestinal tract concerns motility and secretion, regulating muscular peristaltic activity [46] and being directly implicated in the pathways mediating the bicarbonate secretion in response to luminal acidification [47] and enhancing water secretion [48]. Interestingly, although NPY was co-localized with 5-HT in neuroendocrine cells of duodenum, the cellular localization appeared different, with NPY mainly localized in the cytoplasm and 5-HT on the cell membrane. This pattern was also confirmed in the positive control. At intestinal levels, NPY acts in autocrine or paracrine manner to regulate all aspects of nutrient homeostasis including satiety, mechanical and chemical digestion, nutrient absorption, storage and utilization [49]. In addition, we observed NPY immunoreactivity in glucagon cells of pancreatic islets, further confirmed by immunofluorescence experiments which excluded co-presence of NPY with 5-HT, known to be marker of insulin cells [50,51]. 5-HT is synthesized by β-cells and promotes their functions [52], regulating insulin secretion as local autocrine/paracrine signal [53] and inhibiting glucan secretion in paracrine manner [50]. The localization of NPY in the pancreas is still matter of debate in mammalian pancreas. In very close species to goat, immunoreactivity to NPY was appreciated in nerve fibers in the endocrine pancreas of calf and cow [54], and in neurons of endocrine pancreas of sheep [55]. Further studies from animal models have concluded that NPY is primarily expressed in β-cells of murine models [56,57,58] while others have reported that it is found in the glucagon secreting alpha cells [59] or in the somatostatin-containing delta cells [60]. A most recent study suggests that NPY-immunoreactivity reported in alpha and delta cells from other studies was likely due to the presence of NPY-related [61]. The antibody we employed here is raised against NPY, and we thus exclude cross-reaction with other peptides of the NPY family. The direct effect of NPY on the endocrine pancreas is to suppress insulin via autocrine and/or paracrine mechanisms [62] and stimulate glucagon secretion [63]. Very interestingly, the pattern of NPY distribution in all analysed gastro–entero–pancreatic tracts changed drastically in RLE fed goats, differently from data reported in the hypothalamus of rat fed with ANTs, where levels of NPY were decreased with a consequent effect on lipogenesis [24]. These differences may be due to the percentage of ANTs contents in the feed supplementation of the two experimental design. In our study, a statistically significant higher number of NPY immunoreactive cells was observed in the abomasal epithelium and in the pancreatic islets but not in the duodenum. The remarkable difference observed between abomasum and duodenum could be ascribed to the pH variation of the two environments, which affect the chemistry and the availability of compounds rich of FLAVs, ANTs and other polyphenols [64]. ANTs are indeed pH sensitive [38]. ANTs are reported also to influence and increase insulin secretion in pancreatic β-cells, as demonstrated in vitro studies [65]. In goat kids, we observed an increase of NPY pancreatic α-cells and we suspect a different metabolic regulation of ANTs and NPY, likely due to evolutionary adaptation and feeding habits of the species. Future experiments, also in closed related species, may be helpful to clarify this aspect.

## 5. Conclusions

The effect of feeding supplementation of RLE extract rich in FLAVs, ANTs and other polyphenols on the increase of NPY at gastro-intestinal levels opens new avenues for future studies on the metabolic effect of ANTs and could support the use of natural compounds as alternative feeding resources to ameliorate animal and human health. Furthermore, the peripheral increase of NPY associated to RLE supplementation represents the baseline for developing more accurate experiments to investigate the interactions between NPY and ANTs, in addition to provide a complete characterization of meat quality and animal performance of goat kids fed with RLE used as feed supplementation.

## Figures and Tables

**Table 1:** Chemical composition of red orange and lemon extract (RLE) used as food supplement for the study.

Class of Compounds	Relative Composition (%)
Total flavanones	15.91 ± 0.01
Total Anthocyanins (as cyanidin 3-glucoside equivalents)	2.66 ± 0.01
Total Hydroxycinnamic acids	1.77 ± 0.02
Ascorbic acid	2.40 ± 0.01

## Data Availability

Datasets used in the analyses are stored at the authors’ home institution and will be provided upon request.

## Supplementary Materials

The following are available online at https://www.mdpi.com/2076-2615/11/2/449/s1, Figure S1: NPY and 5-HT immunoreactivity in the duodenum of mouse used as positive control. (a) NPY mainly localized in cells of basal epithelial lamina. (b) 5-HT localized in epithelial cells of intestinal villi. (c) Co-staining of NPY/5-HT in enteroendocrine cells of duodenum. Scale bar: a = 100 µm; b = 50 µm; b_1_-c = 25 µm.
