# Supplementary material for: Distinct Pattern of NPY in Gastro–Entero–Pancreatic System of Goat Kids Fed with a New Standardized Red Orange and Lemon Extract (RLE)
Source: Animals (Basel). 2021 Feb 9;11(2):449. doi: 10.3390/ani11020449 (PMC7914828; doi:10.3390/ani11020449)
Supplement: Supplementary file 1 [file animals-11-00449-s001.pdf]

Distinct pattern of NPY in gastro-entero-pancreatic system of goat kids fed with a new standardized red orange and lemon extract (RLE)

Elena De Felice, Daniela Giaquinto, Sara Damiano, Angela Salzano, Simona Fabroni, Roberto Ciarcia, Paola Scocco, Paolo de Girolamo and Livia D'Angelo

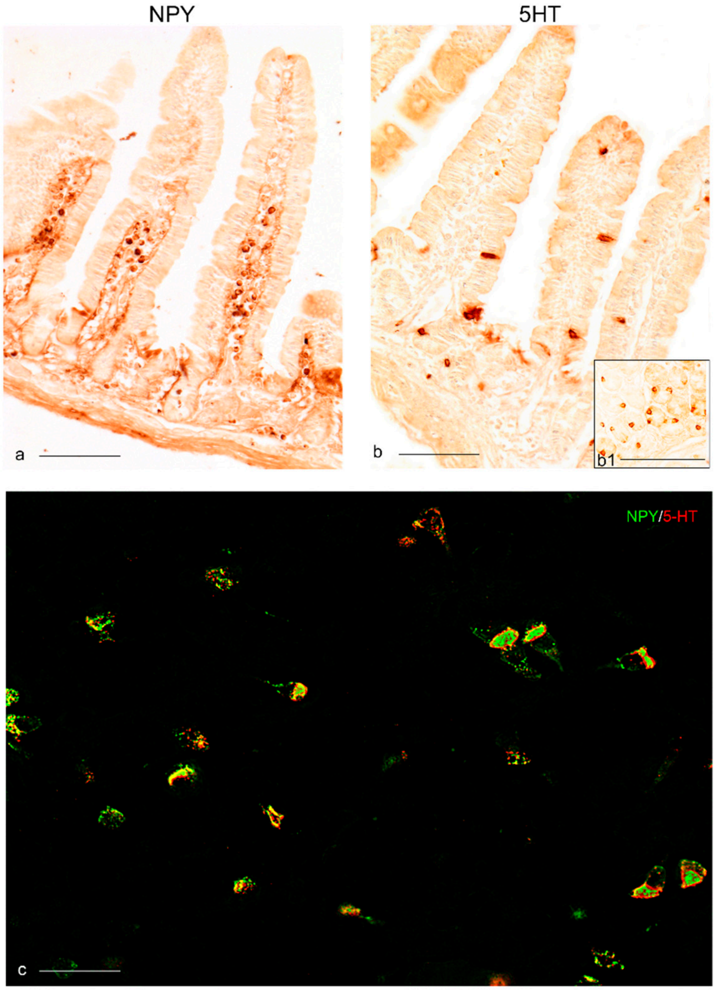

Figure S1. NPY and 5-HT immunoreactivity in the duodenum of mouse used as positive control. (**a**) NPY mainly localized in cells of basal epithelial lamina. (**b**) 5-HT localized in epithelial cells of intestinal villi. (**c**) Co-staining of NPY/5-HT in enteroendocrine cells of duodenum. Scale bar: a = 100μm; b = 50μm; b_1-c = 25μm.
